# Full‐Spectrum Mechanochromic Photonic Films with Large Interparticle Distance

**DOI:** 10.1002/advs.202413881

**Published:** 2025-01-07

**Authors:** Hwan‐Young Lee, Jun‐Gu Kang, Young‐Seok Kim, Shin‐Hyun Kim

**Affiliations:** ^1^ Department of Chemical and Biomolecular Engineering Korea Advanced Institute of Science and Technology (KAIST) Daejeon 34141 Republic of Korea; ^2^ Korea Electronics Technology Institute (KETI) Seongnam Gyeonggi‐do 13509 Republic of Korea

**Keywords:** mechanochromism, photonic bandgap, photonic crystals, structure colors, swelling

## Abstract

Non‐close‐packed crystalline arrays of colloidal particles in an elastic matrix exhibit mechanochromism. However, small interparticle distances often limit the range of reversible color shifts and reduce reflectivity during a blueshift. A straightforward, reproducible strategy using matrix swelling to increase interparticle distance and improve mechanochromic performance is presented. Photonic composites are initially prepared with silica particle arrays embedded in an elastomer matrix at volume fractions of 0.35–0.5. To increase interparticle distance, the composites are immersed in an elastomer‐forming monomer, causing the matrix to swell, followed by photopolymerization, thereby producing liquid‐free composites. The degree of swelling is controllable up to 3.16, depending on monomer choice, matrix volume fraction, and crosslinking density. The process can be repeated to further increase swelling up to 10.36. This method can reduce the volume fraction of silica particles from 40% to 3.8%, while interparticle distance increases from 53 to 257 nm. The swollen photonic composites exhibit a full visible spectrum under compression, while minimizing reflectivity loss. This allows red‐colored photonic composites to be transformed into vivid multicolor patterns when compressed with stamps featuring spatial height variations.

## Graphical abstract

Elastic photonic composites with non‐close‐packed colloidal particle arrays are created to provide full‐spectrum mechanochromism with minimal reflectivity loss. Swelling the elastic matrix of colloidal arrays with elastomer‐forming monomers increases interparticle distance, followed by photopolymerization to stabilize the structure. These liquid‐free films transform into vivid multicolor patterns when compressed using 3D stamps with spatial height variations.

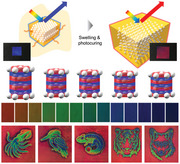

## 1 Introduction

Colloidal crystals and their derivatives, such as inverse opals, display structural colors through the wavelength‐selective reflection of visible light by photonic stopbands.^[^ ^1^, ^2^, ^3^, ^4^, ^5^ ^]^ Unlike chemical pigments, these structural colors are iridescent, nonfading, nontoxic, and tunable, making them highly promising for various optical applications.^[^ ^6^, ^7^, ^8^, ^9^, ^10^, ^11^ ^]^ Notably, photonic structures crafted from elastic materials can dynamically and reversibly change color in response to mechanical deformation, a phenomenon known as mechanochromism.^[^ ^12^, ^13^, ^14^, ^15^, ^16^, ^17^ ^]^ This property has potential applications in areas including colorimetric strain or stress sensors, wearable displays, and military camouflage.

The panther chameleon, which utilizes mechanochromic iridophores containing non‐close‐packed regular arrays of guanine nanocrystals, dynamically controls its skin color by contracting or expanding these cells.^[^ ^18^, ^19^, ^20^ ^]^ Inspired by this natural mechanism, artificial mechanochromic photonic structures have been engineered with a non‐close‐packed array of particles embedded in an elastic matrix.^[^ ^21^, ^22^, ^23^, ^24^, ^25^ ^]^ The spacing between inelastic particles in non‐close‐packed arrays allows them to rearrange freely during macroscopic deformation without disrupting neighboring particles, all while preserving their crystalline order, whereas particles in close‐packed arrays are interlocked, limiting their ability to rearrange. For instance, colloidal particles consisting of an inelastic polymer core and elastic polymer shells are self‐assembled into crystalline structures through thermal compression.^[^ ^24^, ^26^, ^27^, ^28^ ^]^ This process fuses the shells together, forming non‐close‐packed arrays of cores within an elastic matrix. Alternatively, silica particles are assembled into non‐close‐packed arrays within a photocurable resin that transforms into an elastomer through photopolymerization.^[^ ^25^, ^29^, ^30^, ^31^, ^32^, ^33^ ^]^ Here, a solvation layer on the particle surfaces induces repulsion, leading to spontaneous crystallization at relatively low volume fractions.^[^ ^34^, ^35^ ^]^

Achieving a wide range of color change, high reversibility, and linear response requires a significant distance among the inelastic particles. However, conventional approaches offer limited control over interparticle distance despite being suitable for scalable production, leading to a notable reduction in reflection intensity at the mechanically shifted stopband.^[^ ^25^, ^29^, ^30^, ^31^, ^32^ ^]^ Efforts to increase interparticle distance have been made.^[^ ^24^, ^25^, ^33^ ^]^ For example, colloidal particles with a zinc sulfide core and a thick silica shell are assembled into a close‐packed array by dip‐coating, of which the interstitial voids are initially filled with elastomer, and after selectively etching out the silica shells, the voids are filled again with elastomer.^[^ ^25^ ^]^ Despite achieving large interparticle distance and high color tunability, the complexity and low throughput of such processes limit their practical applications.

In this study, we introduce a facile swelling method to produce solvent‐free photonic composites with enhanced interparticle distance, achieving full visible spectrum mechanochromism with improved reflectivity. Initially, elastic photonic composites with relatively short interparticle distances are prepared using silica particles dispersed in an elastomer‐forming acrylate monomer. These composites are then immersed in additional elastomer‐forming acrylate monomers to induce swelling, significantly expanding the lattice constant while maintaining high reflectivity at the red‐shifted stopband, which is stably captured by photopolymerization without residual liquid components. The swelling ratio is adjustable, depending on the choice of elastomer‐forming acrylate monomer, the initial volume fraction of the elastic matrix, and the crosslinking density. Furthermore, the photonic composites can undergo additional swelling and photopolymerization steps to further increase the interparticle distance. With this multiple swelling strategy, it is possible to produce stopbands in the infrared using silica particles with diameters as small as 150 nm. The resulting photonic composites exhibit a broader range of compression‐induced blueshifts while maintaining higher reflectivity compared to those without lattice expansion. Red photonic composite films can be transformed into multicolor patterns spanning the full rainbow spectrum by compressing them using 3D stamps with spatial height variations.

## 2 Results and Discussion

### 2.1 Swelling Photonic Composites with Various Elastomer‐Forming Monomers

**Figure 1:**
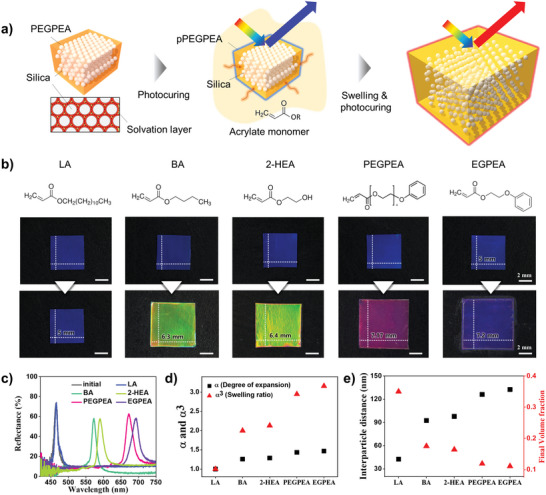
Swelling elastic photonic crystals with elastomer‐forming monomers. a) Schematic illustration of the swelling process in elastic colloidal crystals using acrylate monomers, followed by photopolymerization to create solvent‐free elastic photonic crystals with increased interparticle distance. b) Molecular structures of the monomers used, along with images of the pristine photonic film and the corresponding swollen films. The monomers include lauryl acrylate (LA), butyl acrylate (BA), 2‐hydroxyethyl acrylate (2‐HEA), poly(ethylene glycol) phenyl ether acrylate (PEGPEA), and ethylene glycol phenyl ether acrylate (EGPEA). The dimensions of the films are indicated. c) Reflectance spectra comparing the pristine film with the swollen films produced using various monomers. d) Degree of expansion (*α*) and swelling ratio (*α* ^3^), calculated from the reflectance peak positions for different monomers. e) Interparticle distance and final volume fraction of particles in the swollen films for each monomer.

Elastic photonic composites were produced by dispersing monodisperse silica particles in an elastomer‐forming monomer, specifically poly(ethylene glycol) phenyl ether acrylate (PEGPEA), above the threshold volume fraction in the presence of a photoinitiator. The acrylate groups in PEGPEA form hydrogen bonds with the silanol groups on the surfaces of silica particles. These surface‐bound PEGPEA molecules further interact with additional PEGPEA molecules, forming a thick solvation layer that stabilizes the dispersion, as shown in Figure S1a (Supporting Information).^[^ ^34^, ^35^ ^]^ When the volume fraction of silica particles is sufficiently high, these solvation layers overlap, leading to the spontaneous formation of non‐close‐packed crystalline arrays with a face‐centered cubic (fcc) lattice structure, as illustrated in **Figure** **1**a.

The solvation layer thickness for PEGPEA was estimated at 36.5 nm based on the crystallization behavior as a function of volume fraction.^[^ ^32^ ^]^ Assuming this thickness is independent of the silica particle diameter, the threshold volume fraction, *ϕ*_th_, responsible for spontaneous crystallization was calculated for a given silica particle diameter, *D* (see Figure S1, Supporting Information):

(1) $$\;\phi_{th}=\frac{\pi }{3\sqrt{2}}{\left(\frac{D}{D+73}\right)}^{3}$$

Using monodisperse silica particles with a diameter of *D* = 150 nm, photonic composite films were prepared, setting the volume fraction at *ϕ* = 0.35, which exceeded the calculated threshold, *ϕ*_th_ = 0.225. The diameter of the silica particles exhibited a coefficient of variation (CV) of 4%, as illustrated in Figure S2 (Supporting Information). The dispersion was confined between two glass slides separated by 120 µm, and the top slide was pulled at a constant speed of 100 µm s^−1^ for 125 s to induce shear flow, enhancing the crystalline order of the silica particles.^[^ ^10^ ^]^ Following this, the dispersion was exposed to ultraviolet (UV) light to initiate radical polymerization, covalently linking the acrylate groups of PEGPEA molecules. Since PEGPEA contained a single acrylate group, the polymerized PEGPEA formed linear chains without chemical crosslinking. This polymerization transformed the liquid dispersion into a solid photonic composite film, which displayed a vivid blue coloration.

To further increase the interparticle distance, the films were immersed in various elastomer‐forming monomers. To avoid residual liquid components in the final composites, only acrylate‐based monomers were used without any organic solvents. These included lauryl acrylate (LA) with 1% crosslinker of poly(ethylene glycol) diacrylate (PEGDA), butyl acrylate (BA) with 1% PEGDA, 2‐hydroxyethyl acrylate (2‐HEA), PEGPEA, and ethylene glycol phenyl ether acrylate (EGPEA); all monomers contained 1% photoinitiator for photopolymerization. LA and BA required crosslinkers to form solid elastomers, whereas 2‐HEA, PEGPEA, and EGPEA formed elastomers without crosslinkers. After a one‐day incubation period, the films were recovered and UV‐irradiated to produce solvent‐free photonic composites with an expanded lattice, as depicted in Figure 1a.

Swelling of the photonic composite films resulted in film expansion and a redshift in the structural colors, as illustrated in Figure 1b. The degree of expansion varied depending on the elastomer‐forming monomer used. For instance, films immersed in LA showed no significant change in size or color, while those immersed in BA, 2‐HEA, PEGPEA, and EGPEA exhibited considerable expansion and color changes. Specifically, the films changed from blue to green in BA and 2‐HEA, with lateral dimension increases of 1.26 and 1.28 times, respectively. In PEGPEA and EGPEA, the dimensions increased by factors of 1.43 and 1.47, with the colors shifting to magenta and violet, respectively.

The structural color changes were further analyzed using reflectance spectra, as shown in Figure 1c. The pristine photonic films exhibited a reflectance peak at *λ*_peak_ = 465 nm, consistent with the diffraction wavelength *λ*_111_ = 465 nm, predicted by Bragg's law for the (111) plane of the fcc lattice:

(2) $$\;\lambda_{111}=2d_{111}n_{eff}={\left(\frac{\pi }{3\sqrt{2}\phi }\right)}^{1/3}{\left(\frac{8}{3}\right)}^{1/2}\;D{\left(n_{silica}^{2}\phi +n_{PEGPEA}^{2}\left(1-\phi \right)\right)}^{1/2}$$

where *d*_111_ represents the interplane distance, and *n*_eff_ is the effective refractive index.^[^ ^36^ ^]^ The effective refractive index is approximated from the refractive indices of silica and PEGPEA, *n*_silica_ = 1.45 and *n*_PEGPEA_ = 1.502. These results confirmed that the photonic films consisted of (111) plane stacks along the thickness direction. The absence of swelling in the LA‐immersed films was corroborated by the unchanged reflectance peak. In contrast, the peak significantly redshifted for all other monomers. As swelling increased the lattice constant by expanding the PEGPEA matrix without significantly affecting the silica particles, the Bragg equation was modified as follows (see Figure S3, Supporting Information):

(3) $$\;\lambda_{111}=2d_{111}n_{eff}=\alpha {\left(\frac{\pi }{3\sqrt{2}\phi }\right)}^{1/3}{\left(\frac{8}{3}\right)}^{1/2}D{\left(n_{silica}^{2}\frac{\phi }{\alpha^{3}}+n_{mixture}^{2}\left(1-\frac{\phi }{\alpha^{3}}\right)\right)}^{1/2}$$

where *α* (= *a*_f_/*a*_i_) denotes the degree of lattice expansion, *α* ^3^ represents a swelling ratio, and *n*_mixture_ is the refractive index of polymer matrix formed by polymerizing monomer‐swollen pPEGPEA. The volume fraction of silica particles in swollen matrix is *ϕ*/*α* ^3^. The lattice expansion degree, estimated as *a*_f_/*a*_i_ = 1.26 from 𝜆_peak_ = 573 nm for BA, aligned with the lateral expansion of 1.26 times observed in the bulk film, indicating isotropic expansion of the photonic films. The corresponding swelling ratio was *α* ^3^ = 2.0. For 2‐HEA, PEGPEA, and EGPEA, the reflectance peak positions were at *λ*_peak_ = 591, 672, and 692 nm, with estimated *a*_f_/*a*_i_ values of 1.28, 1.43, and 1.47, respectively. These values were consistent with the observed lateral expansions of the bulk films. The swelling ratios were calculated as *α* ^3^ = 2.13, 2.95, and 3.16, respectively, and are summarized in Figure 1d. Notably, the swelling ratio increased with the molecular similarity and compatibility of the monomers to polymerized PEGPEA. However, the swelling ratio does not directly correlate with the Hansen distance, as specific molecular interactions among various molecular moieties are not fully captured in solubility parameters. The higher swelling ratio for EGPEA compared to PEGPEA was attributed to the smaller molecular size despite comparable molecular similarity.

As the swelling ratio increased, the surface‐to‐surface distance between silica particles within the polymer matrix also increased. For silica particles with a diameter of *D* = 150 nm and a volume fraction of *ϕ* = 0.35 in PEGPEA, the initial distance was 42 nm. This distance increased to 92 nm for *α* ^3^ = 2.0 (BA), 98 nm for *α* ^3^ = 2.13 (2‐HEA), 126 nm for *α* ^3^ = 2.95 (PEGPEA), and 132 nm for *α* ^3^ = 3.16 (EGPEA), as shown on the left y‐axis of Figure 1e. Concurrently, the silica particle volume fraction decreased from *ϕ* = 0.35 for α = 1–0.175 for *α* ^3^ = 2.0 (BA), 0.164 for *α* ^3^ = 2.13 (2‐HEA), 0.118 for *α* ^3^ = 2.95 (PEGPEA), and 0.111 for *α* ^3^ = 3.16 (EGPEA), as illustrated on the right *y*‐axis. To examine the increased distance among silica particles, cross‐sections of unswollen and PEGPEA‐swollen films were analyzed using a scanning electron microscope (SEM), as depicted in Figure S4 (Supporting Information). While it is challenging to determine the precise value of the distance from the images, the lattice expansion is readily apparent. In this study, PEGPEA was predominantly used as the swelling monomer.

### 2.2 Swelling Dynamics of Photonic Films

**Figure 2:**
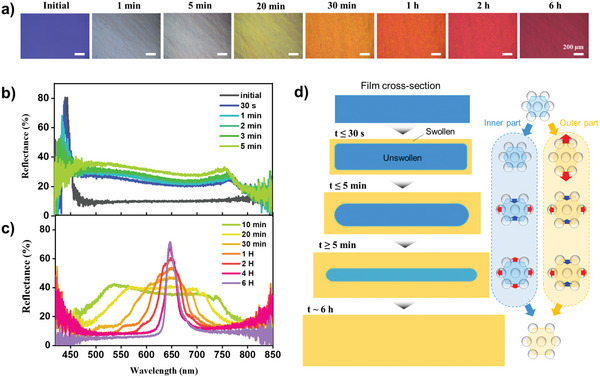
Anisotropic expansion of photonic films during swelling. a) A series of optical microscope (OM) images taken from the central part of the film showing the temporal color change during the swelling by PEGPEA. b,c) Reflectance spectra captured during the swelling process, showing spectra taken up to 5 min (b) and taken after 5 min (c). d) Schematic illustration depicting the swelling dynamics of the thin film, highlighting the lattice deformation occurring in the inner and outer regions of the film. The duration of each step is denoted based on the temporal changes observed in the reflectance spectra.

When the photonic films were submerged in monomers, they underwent swelling, ultimately leading to isotropic expansion, as illustrated in Figure 1. However, during the swelling process, the films exhibited unexpected color changes and anisotropic expansion, as shown in **Figure** **2**a and Movie S1 (Supporting Information), where the blue film, produced from silica particles with a diameter of *D* = 150 nm and the volume fraction at *ϕ* = 0.40, was used. The blue film with a thickness of 120 µm and lateral dimensions of 5 × 5 mm^2^ turned colorless within the first 5 min, during which lateral expansion was minimal. Subsequently, the film gradually expanded laterally over the course of 6 h, accompanied by noticeable color changes. This dynamic behavior significantly deviates from the initial expectation of a gradual redshift from blue to red caused by isotropic expansion.

To investigate the swelling behavior further, the reflectance spectrum of the film was monitored in its central region. Initially, the film exhibited a single peak at *λ*_peak_ = 445 nm. Upon submersion in PEGPEA monomer, the peak slightly blueshifted, and its intensity significantly decreased within the first 5 min, as shown in Figure 2b. Simultaneously, a new peak appeared near 770 nm within 30 s, which then blueshifted to 750 nm after 5 min. This blueshift, along with the reduction in intensity of the original blue peak and the emergence of a new peak in the near‐infrared region, rendered the film colorless. Despite this, the silica particles, embedded in a solid pPEGPEA matrix, retained their lattice structure, albeit in a deformed state. Over the following 20 min, the original peak in the blue region redshifted while the peak in the near‐infrared region continued to blueshift, forming a broad plateau. Eventually, the two peaks merged into a single peak near 646 nm within 1 h, which then became sharper and more pronounced after 6 h.

Contrary to the initial expectation of a gradual redshift of the original peak to its final position, the swelling process involved an early stage blueshift of the original peak and the simultaneous emergence of a new peak and its blueshift. Additionally, lateral expansion of the film was minimal during this early stage. These observations suggest that PEGPEA swelled the film primarily along its surfaces, leading to significant lattice expansion in the outer region along the thickness direction. This swelling caused the development of a new peak at a longer wavelength within 30 s. As the inner region of the film remained unswollen due to slow monomer permeation, it restricted the lateral expansion of the outer region. However, as monomer penetration increased, the swollen outer region exerted stress on the inner region, causing slight lateral stretching. This process reduced the film's thickness, leading to a blueshift of the original peak within 5 min. Concurrently, the thickness of the completely unswollen portion decreased, further diminishing the intensity of the blueshifted original peak. The stretching of the inner region reduced the constraints on the lateral expansion of the swollen outer region, which then expanded laterally, reducing anisotropy in the swelling. The weak but noticeable blueshift of the newly developed peak indicates that the lattice in the outer region slightly shrank along the thickness direction while expanding laterally. This early stage swelling behavior, relevant up to 5 min, is depicted in the second and third rows of Figure 2d.

Once the PEGPEA monomers penetrated the middle of the film's cross‐section, lateral expansion was no longer restricted. The inner region of the film expanded significantly in both lateral and thickness directions, while the outer region continued to shrink slightly in thickness during lateral expansion. Ultimately, the swelling became isotropic throughout the film, resulting in a sharp and intense single peak with unified lattice spacing. This uniform expansion behavior was observed even in films with lateral dimensions of several centimeters, as shown in Figure S5 (Supporting Information), confirming that the peculiar swelling behavior was not caused by structural heterogeneity of the silica‐pPEGPEA composites.

The anisotropic swelling behavior can be attributed to the anisotropic geometry of the thin film. Since monomer permeation predominantly occurs along the thickness direction, the outer region swells while the inner region, located in the middle of the cross‐section, retains its volume. The inner region's restriction of the lateral expansion of the outer region amplifies the expansion along the thickness direction during the early stage. Simultaneously, the swelling of the outer region exerts lateral stress on the inner region, causing it to stretch. The diffusion and swelling kinetics of the photonic films at the microscopic level closely align with those observed in polymers, particularly under conditions of slow Case II diffusion.^[^ ^37^ ^]^ However, no prior studies appear to have detailed the anisotropic swelling kinetics of freestanding polymer films with isotropic molecular structures at both macroscopic and microscopic levels. Our analysis, based on the temporal evolution of reflectance spectra combined with direct observation of macroscopic films, reveals that the films initially swell beyond their equilibrium dimensions along the thickness near the surfaces. This occurs because the unswollen inner region resists lateral expansion in the early stages. Subsequently, as swelling progresses in the inner region, lateral expansion is enabled, leading to the relaxation of the initially excessive thickness swelling in the outer regions, ultimately reaching an isotropic equilibrium state.

When a sample was prepared in a cubic shape with dimensions of 3 × 3 × 3 mm^3^ instead of as a film, a completely different behavior was observed, as shown in Figure S6 (Supporting Information); the cubic sample was produced from silica particles with a diameter of *D* = 150 nm and the volume fraction at *ϕ* = 0.35, which results in *λ*_peak_ = 465 nm before swelling. The original peak diminished while a new peak developed. This new peak gradually redshifted to 710 nm and increased in intensity without exhibiting the unexpected blueshift observed in the thin film; the degree of swelling for the cubic sample, estimated from the peak positions before and after swelling, is 3.56, which is higher than that of the film sample, which is 3.06, due to the higher volume fraction of the swellable pPEGPEA matrix. In the cubic sample, monomer permeation occurred from all surfaces at a comparable depth, avoiding directional stress. This behavior also deviates from the initial expectation of a gradual redshift of the original peak to its final position. Instead, the slow diffusion of PEGPEA monomer caused the composite to swell uniformly from the surfaces inward.

### 2.3 Control Over the Swelling Ratio

**Figure 3:**
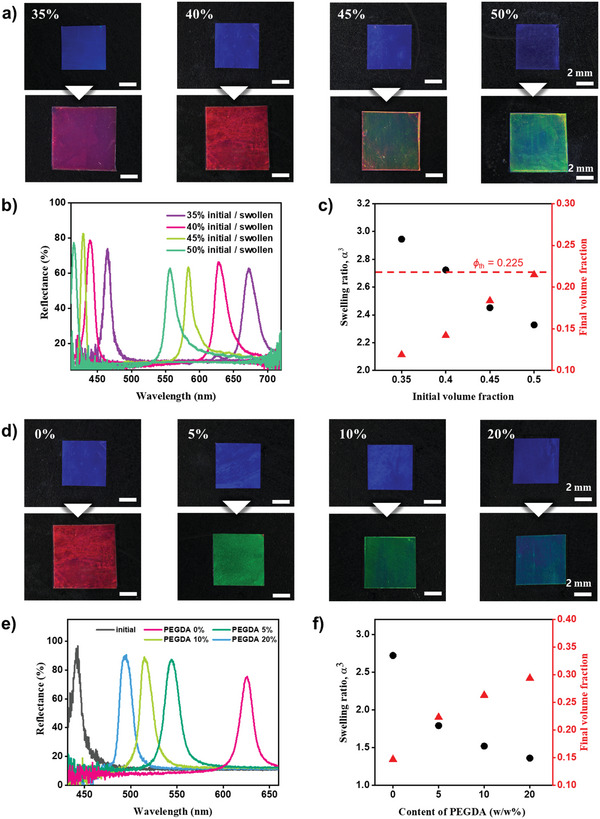
Control over swelling ratio. a,b) Sets of photographs (a) and reflectance spectra (b) of pristine and swollen films with varying initial volume fractions of silica particles, as indicated. c) Swelling ratio and final volume fraction of silica particles in the swollen films as a function of the initial volume fraction. The threshold volume fraction for spontaneous crystallization (0.225) is marked with a horizontal dotted line. d,e) Sets of photographs (d) and reflectance spectra (e) of pristine and swollen films with varying concentrations of the crosslinker PEGDA, as indicated. f) Swelling ratio and final volume fraction of silica particles in the swollen films as a function of PEGDA concentration. PEGPEA is used for swelling for all experiments.

The swelling ratio varied depending on the selected monomer, as shown in Figure 1. For a given monomer, the swelling ratio changed with the volume fraction of silica particles. Since monomers selectively swelled the pPEGPEA matrix without affecting the silica particles, a higher swelling ratio was observed at lower silica particle volume fractions. To study this influence, we prepared four films of identical thickness of 120 µm but with varying particle volume fractions of *ϕ* = 0.35, 0.4, 0.45, and 0.5, all of which exceed the threshold volume fraction for spontaneous crystallization, *ϕ*_th_ = 0.225 for *D* = 150 nm. The films displayed a bluish color across all four volume fractions, as shown in the top panels of **Figure** **3**a. The reflectance spectra exhibited pronounced peaks at *λ*_peak_ = 465, 445, 427, and 412 nm, respectively, as shown in Figure 3b. These peak positions aligned with Equation (2), corresponding to *λ*_111_ = 465, 445, 427, and 412 nm. Since interparticle distance decreased with higher volume fractions, the reflectance peak shifted to shorter wavelengths.

The four films were submerged in PEGPEA for 1 day to achieve full swelling and were then photopolymerized. The films underwent expansion and color change, as depicted in the bottom panel of Figure 3a. Notably, films with lower volume fractions expanded more. To characterize the swelling ratio, the reflectance spectra of the swollen films were obtained, as shown in Figure 3b. The peak positions of the swollen films shifted to *λ*_peak_ = 672, 626, 584, and 556 nm. The peak shift was more significant for films with lower volume fractions. Using the values of *λ*_peak_ for the original and swollen films, the swelling ratio and final volume fractions were calculated using Equation (3). The swelling ratio was 2.33 for *ϕ* = 0.5, increasing to 2.95 for *ϕ* = 0.35, as shown in Figure 3c. The final volume fraction decreased from 0.21 for *ϕ* = 0.5–0.12 for *ϕ* = 0.35. Achieving these final volume fractions without swelling was challenging, as they were lower than the threshold volume fraction, *ϕ*_th_ = 0.225. When considering the volume of the pPEGPEA matrix excluding the silica particles, the swelling ratio remained consistent at 3.9, regardless of the initial volume fraction, confirming that only the pPEGPEA matrix swelled.

As expected, the size of the silica particles did not affect the swelling ratio. To verify this, four photonic films were prepared with silica particles of average diameters *D* = 150, 185, and 200 nm, respectively, while maintaining a volume fraction of *ϕ* = 0.40, as shown in Figure S7 (Supporting Information). Swelling caused the films to expand, leading to a redshift in reflectance peaks from which the swelling ratio and final volume fractions were calculated. The swelling ratio was ≈2.7, and the final volume fraction was 0.14 for all films.

An alternative method to control the swelling ratio was to crosslink the pPEGPEA matrix and adjust the crosslinking density. Since PEGPEA contains a single acrylate group, pPEGPEA forms a linear polymer with side chains. To chemically crosslink pPEGPEA, PEGDA, which has two acrylate groups, was used as a crosslinker. The crosslinking density was adjusted by controlling the mixing ratio of PEGDA relative to PEGPEA. Without PEGDA, the photonic film with *ϕ* = 0.4 changed color from blue to red while significantly expanding upon swelling, as shown in the first column of Figure 3d. When the PEGDA ratio was set to 5:95, the blue film turned green with reduced expansion, as shown in the second column. For higher PEGDA contents of 10:90 and 20:80, the final colors were less redshifted, and the films exhibited less expansion. The reflectance spectra quantitatively demonstrated the redshifts, as shown in Figure 3e. The peak was located at 445 nm for all four films, shifting to 626 nm for 0% PEGDA, 545 nm for 5%, 515 nm for 10%, and 494 nm for 20%. From the peak shifts, the swelling ratios were calculated using equation (3), as shown in Figure 3f. The swelling ratio was 2.72 for 0% PEGDA, decreasing to 1.36 for 20%, at which the final volume fraction of silica particles changed from 0.142 for 0% to 0.294 for 20%. Therefore, the degree of lattice expansion was controlled by adjusting the crosslinking density as well as the initial volume fraction of silica particles.

**Figure 4:**
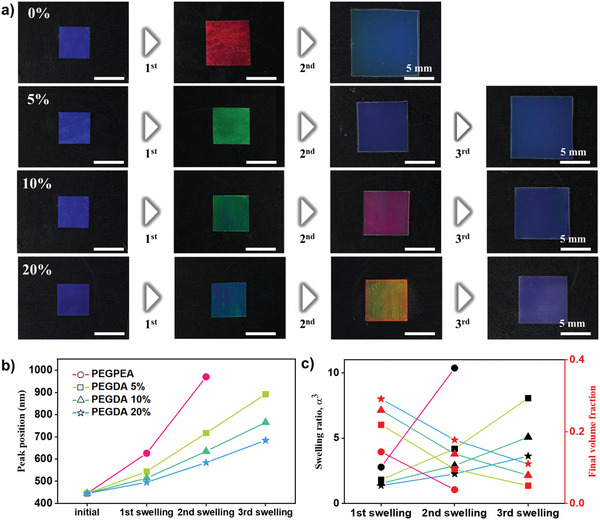
Multiple steps of swelling and photopolymerization. a) Sets of photographs showing the color change and expansion of photonic films during multiple steps of swelling and photopolymerization. The process is shown for photonic films with four different PEGDA concentrations: two steps for 0% PEGDA and three steps for 5%, 10%, and 20% PEGDA. b) Shift in the reflectance peak wavelength during the multiple steps of swelling and photopolymerization for photonic films with the four different PEGDA concentrations. c) Swelling ratio and volume fraction of silica particles as a function of the number of swelling and photopolymerization steps. PEGPEA is used for swelling in all steps.

The photonic films underwent a second round of swelling and polymerization to further increase interparticle distance. For example, the blue photonic film without PEGDA turned red with volume expansion after the first round of swelling and polymerization, and it underwent further significant expansion in the second round, as shown in the top row of **Figure** **4**a. The lateral dimension of the film increased by 2.2 times through two consecutive steps of swelling and polymerization, indicating that the swelling ratio was as high as 10.6. Photonic films with a crosslinked pPEGPEA matrix also demonstrated multi‐step expansions through swelling and polymerization, as shown in the second to fourth rows. During three rounds of swelling and photopolymerization, the films continued to expand.

The reflectance spectra revealed the peak shifts during the multiple steps, as shown in Figure S8 (Supporting Information). The reflectance intensity decreased as the films expanded because the refractive index contrast between the particle‐laden layer and the particle‐free layer in the composites decreased, as shown in Figure S9 (Supporting Information). The redshift of the peak position for films with four different crosslinking densities was summarized in Figure 4b. The peak originally at 445 nm shifted into the near‐infrared region through the visible range. Additionally, the swelling ratio relative to the original state and the volume fraction of silica particles after the first, second, and third rounds of swelling and polymerization were calculated, as shown in Figure 4c. The swelling ratio reached up to 10.36, and the volume fraction decreased from the original value of 0.40–0.038 for highly expanded films. It is noteworthy that the peak position of 970 nm was achieved with silica particles as small as *D* = 150 nm by increasing the interparticle distance as large as 257 nm at the final volume fraction of 0.038 from 53 nm at the initial volume fraction of 0.40. Therefore, consecutive steps of swelling and polymerization provided an effective method to increase interparticle distance in composites.

### 2.4 Mechanochromism and Multicolor Patterning

Swelling increased the interparticle distance between inelastic silica particles, enhancing the wavelength range of mechanochromism. To demonstrate this, photonic films were prepared with a particle diameter of *D* = 150 nm at an initial volume fraction of *ϕ* = 0.40. These films were then subjected to swelling in PEGPEA and photopolymerization, resulting in a red color with a reflectance peak at 626 nm and a final volume fraction of *ϕ* = 0.14. To compare mechanochromism quantitatively with unswollen samples, photonic films were also prepared with *D* = 210 nm at the same initial volume fraction of *ϕ* = 0.40, ensuring a reflectance peak at 626 nm. Both films were compressed using a pair of glasses, with the photonic films placed inside thick polydimethylsiloxane (PDMS) to facilitate compression; direct compression of the photonic films with glasses was challenging due to strong adhesion of the films to the rigid glass surfaces.

**Figure 5:**
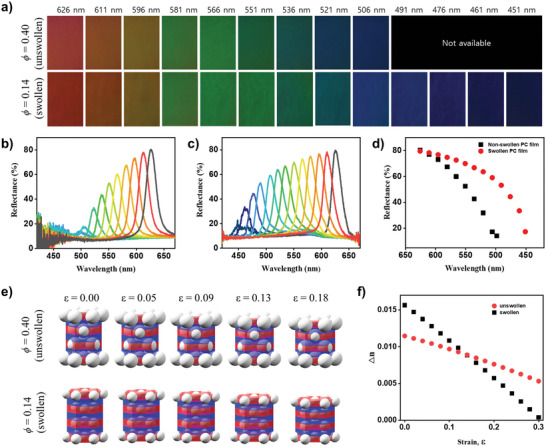
Full‐spectrum mechanochromism. a) Series of OM images showing the compression‐induced color change in an unswollen photonic film with silica particles of *D* = 210 nm at a volume fraction of *ϕ* = 0.40 (top), and a swollen film with silica particles of *D* = 150 nm at a final volume fraction of *ϕ* = 0.14 (bottom). b,c) Series of reflectance spectra recorded during the compression of the unswollen (b) and swollen (c) photonic films. d) Reflectance peak intensity as a function of peak wavelength for the swollen and unswollen films under compression. e) Lattice models depicting the structural changes in the unswollen and swollen films under various strains, as indicated. f) Refractive index contrast between particle‐rich and particle‐poor layers as a function of strain for the unswollen and swollen lattices.

During compression, the structural color shifted from red to blue, and the original red color was reversibly recovered upon relaxation, as shown in Movie S2 (Supporting Information). The compression‐induced color and reflectance spectrum changes were compared between the two films. The unswollen film with *D* = 210 nm and *ϕ* = 0.40 showed a consistent reduction in color brightness and reflectivity during the blueshift from 626 nm, as illustrated in **Figure** **5**a,b. The structural color was lost, and the reflectance peak diminished when compressed beyond 506 nm, corresponding to a compressive strain of 0.19. In contrast, the swollen film with *D* = 150 nm and final *ϕ* = 0.14 exhibited a blueshift down to 451 nm with a compressive strain of 0.28, as shown in Figure 5c. Notably, the swollen film maintained much higher color brightness, particularly in the green and blue regions, as depicted in Figure 5a. This behavior was attributed to the slower reduction of reflectivity in the swollen films, as shown in Figure 5d.

The broader range of color change and the reduced reflectivity loss in the swollen film resulted from the larger distance between the inelastic silica particles. The reflectance peak arose from a stack of hexagonal arrays along the (111) direction of the fcc lattice, as described by Equations (2) and (3). This stacking created a periodic refractive index contrast between the particle‐rich layer, containing the hexagonal array, and the particle‐poor layer, represented by the red and blue regions in Figure 5e. Upon compression, particles in adjacent hexagonal layers gradually interpenetrate, reducing the refractive index contrast between the particle‐rich and particle‐poor layers and, consequently, decreasing reflectivity. Lattice deformation under compressive strain while conserving lattice volume was modeled, as shown in Figure 5e. The difference in effective refractive indices between the particle‐rich and particle‐poor layers was calculated, as depicted in Figure 5f. For the swollen lattice with *D* = 150 nm and *ϕ* = 0.14, the initial index contrast was slightly lower than that of the unswollen lattice with *D* = 210 nm and *ϕ* = 0.40 due to the lower silica particle fraction in the particle‐rich layer. However, the contrast decreased more gradually with strain as the particles were separated by ≈105 nm, leading to a slower reduction in reflectivity. Conversely, the unswollen lattice with *D* = 210 nm and *ϕ* = 0.40, with a shorter interparticle distance of 47 nm, exhibited a steeper decrease in index contrast under strain. The contrast approached nearly zero at a strain of 0.30, leading to a more rapid reduction in reflectivity.

The compression‐induced blueshift and the subsequent relaxation‐induced recovery of structural color in our films are highly reversible. Specifically, the swollen film exhibited consistent spectra under a compressive strain of 0.20 and at a stress‐free zero strain state across 20 compression and relaxation cycles, as depicted in Figure S10 (Supporting Information). The spectral shape, peak wavelengths, and reflectivity remained unchanged in both states throughout these cycles. Although it was initially anticipated that swelling would reduce the modulus due to a decrease in particle volume fraction, our measurements indicated the opposite: the modulus actually increased post‐swelling, particularly for the compressive strain higher than 0.10, as shown in Figure S11 (Supporting Information). This phenomenon likely arises because the physically entangled pPEGPEA chains undergo partial stretching during the swelling process, which contributes to the increased stiffness of the films.

**Figure 6:**
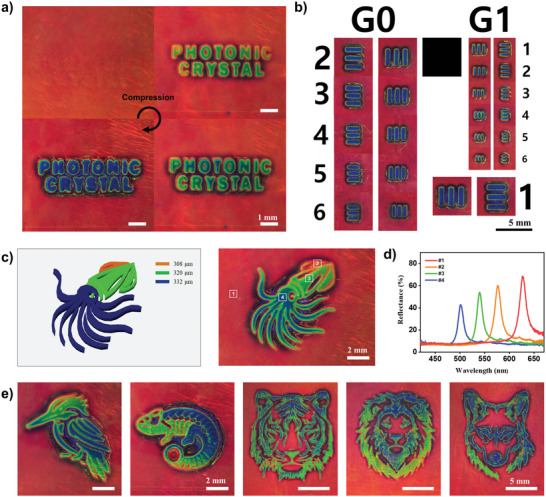
Development of color patterns through stamping. a) OM images displaying the photonic film in its uncompressed state (top‐left) and progressively compressed states (clockwise) using a stamp with the text “PHOTONIC CRYSTAL”. The degree of compression is increased sequentially. b) Collection of OM images showcasing local color changes in the photonic film when compressed with stamps featuring Elements 1–6 from Groups 0 and 1 of the USAF 1951 resolution chart. c) Design of a squid‐shaped stamp with three distinct height levels (indicated in the first panel) and OM image of the photonic film after compression with this stamp. d) Reflectance spectra taken at four different positions as denoted in (c). e) OM images showing the color patterns of a bird, chameleon, tiger, lion and wolf, developed through stamping.

The swollen photonic films with enhanced interparticle distance were compressed using positive stamps to induce localized color changes. For instance, when compressed with a stamp featuring the text “PHOTONIC CRYSTAL,” the color of the words transitioned to green and blue, depending on the degree of compression, while the background remained red, as shown in **Figure** **6**a. Due to the elastic nature of the films, this color change was highly reversible, leaving no trace of deformation once the stamp was removed, as shown in Movie S3 (Supporting Information). To assess the mechanochromic resolution of the swollen film, we fabricated stamps featuring Elements 1–6 for Groups 0 and 1 from the USAF 1951 resolution chart, a standard tool for evaluating resolution, using 3D printing. When the swollen films were compressed by these stamps, color changes were observed on the positive patterns of the elements, as shown in Figure 6b. The finest resolved element was Element 6 in Group 1, which displayed a clear color change consistent with the positive pattern. The rounded features and nonuniform widths of the blue lines, as well as the interline separation, resulted from imperfections in the stamps caused by the limited resolution of the 3D printing process. The successful color representation of Element 6 in Group 1 demonstrates that the resolution of the swollen film is at least 3.87 line pairs per millimeter (lp/mm), enabling it to distinctly resolve line spacings of ≈260 µm.

By using 3D stamps with varying spatial heights, the films were transformed into multicolor patterns. For example, a 3D stamp of a squid, with height variations of 332 µm for the arms and tentacles, 320 µm for the head and right fin, and 308 µm for the left fin, transformed the uniform red film into a multicolored squid pattern, as shown in Figure 6c. The arms and tentacles appeared blue with a reflectance peak at 501 nm under the highest strain, the head and right fin showed green with a peak at 539 nm under intermediate strain, and the left fin displayed orange with a peak at 577 nm under the lowest strain, all set against a red background with a peak at 626 nm under zero strain, as shown in Figure 6c,d. Similarly, multicolor graphics of a bird, chameleon, tiger, lion, and wolf were produced using corresponding 3D stamps, as shown in Figure 6e. These multicolor graphics vanished when the stamps were removed and reappeared upon compression in a highly reversible manner.

## 3 Conclusion

In this study, we develop a strategy to increase the interparticle distance of inorganic silica particles embedded in an elastic polymer matrix, enabling full visible spectrum mechanochromism. We prepare elastic photonic films with limited interparticle distance through the spontaneous crystallization of silica particles driven by short‐range repulsion. These films are then subjected to swelling using elastomer‐forming monomers, which are fully cured after swelling to avoid liquid components. The degree of swelling is controllable up to 3.16, depending on the selection of monomers, the initial elastomer volume fraction, and the crosslinking density, all while maintaining high reflectivity. The degree of swelling can be further increased up to 10.36 through additional cycles of swelling and polymerization. This approach positions the photonic stopband at 970 nm in a non‐close‐packed array of 150 nm silica particles, with surface‐to‐surface distance increasing to 257 nm, compared to 53 nm before swelling. The photonic films with expanded lattice structures exhibit a broader range of compression‐induced blueshifts and reduced reflectivity loss compared to unswollen composites with the same stopband position in the unstrained state. These films generate multicolor patterns spanning the entire rainbow spectrum through controlled compression. Compared to prior studies on mechanochromic colloidal photonic crystals, our current approach offers significant advantages, including a larger wavelength shift and a more gradual decrease in reflectivity, as summarized in Table S1 (Supporting Information). We believe these enhanced mechanochromic materials are useful for reflection‐mode color displays, sensors, and anti‐counterfeiting technologies, where mechanical deformation dynamically tunes the reflective color.

## 4 Experimental Section

### Preparation of Elastic Photonic Films

Monodisperse silica particles with average diameters of 150, 185, and 200 nm (Sukgyung AT) were washed with ethanol, dried, and weighed. The dried silica powders were redispersed in ethanol using sonication for 12 h, and PEGPEA (Mn 324 g mol^−1^, Sigma–Aldrich) containing 1 w/w% photoinitiator Darocur 1173 (Sigma–Aldrich) was added to achieve silica particle volume fractions of 0.35, 0.4, 0.45, and 0.5 at ethanol‐free basis, where the densities of silica and PEGPEA were considered as 2.0 and 1.127 g mL^−1^, respectively. Ethanol was evaporated from the dispersion in a convection oven (OF‐22G, Jeio Tech) at 70 °C for 12 h. The silica‐in‐PEGPEA dispersion was placed on a glass slide with 120‐µm‐thick tape spacers (3M) along the edges. Another glass slide was placed on top to spread the dispersion evenly. A constant shear rate of 0.83 s^−1^ was applied by fixing the bottom slide while pulling the top slide using a syringe pump at a rate of 0.1 mm s^−1^ for 125 s.^[^ ^13^ ^]^ The slides were then secured by taping, and the dispersion was exposed to UV light (CoolWave UV Curing System, Nordson) at an intensity of 120 W cm^−^^2^ for 60 s.

### Swelling and Compression of Photonic Films

Photonic films were fully immersed in elastomer‐forming acrylate monomers, including LA (Mw 240.38 g mol^−1^, Sigma–Aldrich) containing 1 w/w% PEGDA (Mn 575 g mol^−1^, Sigma–Aldrich), BA (Mw 128.17 g mol^−1^, Sigma–Aldrich) containing 1 w/w% PEGDA, 2‐HEA (Mw 116.12 g mol^−1^, Sigma–Aldrich), PEGPEA, and EGPEA (Mw 192.21 g mol^−1^, Sigma–Aldrich). All monomers contained 1 w/w% Darocur 1173. After 24 h of incubation, residual surface monomers were removed by blading with a glass slide. The films were polymerized under UV light for 60 s. To induce color change on entire surface, the films were compressed using a pair of glasses, with the photonic films placed inside 360‐µm‐thick PDMS layers. For regioselective color modulation, various stamps with uniform height (PHOTONIC CRYSTAL pattern, resolution test chart) or spatial height variations (animal patterns), made using a 3D printer (MIICRAFT Plus, MIICRAFT), were used without PDMS layers.

### Characterization

Photonic films were observed using an optical microscope in reflection mode (Eclipse L150, Nikon) and a stereo microscope (SMZ745T, Nikon). Reflectance spectra were measured with a fiber‐coupled spectrometer (USB 4000, Ocean Optics Inc.), integrated with the optical microscope, using a 10× objective lens. A broadband dielectric mirror (BB3‐E02, Thorlabs) was used as the reflectance reference, which exhibits an average reflectivity of 99% in the visible range. The cross‐sections of the photonic films were observed using SEM (SU‐8230, Hitachi) after coating with platinum. Stress‐strain curves were measured three times and averaged using a universal testing machine (MCT‐1150, AND Inc.), where pristine films with a thickness of 120 µm and swollen films with a thickness of 170 µm were used for these tests. The films, placed on glass slides, were compressed at a constant strain rate of 20 mm min^−1^ using a cylindrical probe with a slightly rounded tip and a diameter of 2.3 mm.

## Conflict of Interest

The authors declare no conflict of interest.

## Supporting information



Supporting Information

Supplemental Movie 1

Supplemental Movie 2

Supplemental Movie 3

## Data Availability

The data that support the findings of this study are available in the supplementary material of this article.
