# Supplementary material for: Full‐Spectrum Mechanochromic Photonic Films with Large Interparticle Distance
Source: Adv Sci (Weinh). 2025 Jan 7;12(8):2413881. doi: 10.1002/advs.202413881 (PMC11848551; doi:10.1002/advs.202413881)
Supplement: Supplementary file 1 — Supporting Information [file ADVS-12-2413881-s003.pdf]

ADVANCED SCIENCE
Open Access

Supporting Information

for *Adv. Sci.*, DOI 10.1002/advs.202413881

Full-Spectrum Mechanochromic Photonic Films with Large Interparticle Distance

*Hwan-Young Lee, Jun-Gu Kang, Young-Seok Kim and Shin-Hyun Kim**

Supporting Information

Full-Spectrum Mechanochromic Photonic Films with Large Interparticle Distance

*Hwan-Young Lee, Jun-Gu Kang, Young-Seok Kim and Shin-Hyun Kim**

Department of Chemical and Biomolecular Engineering,
Korea Advanced Institute of Science and Technology (KAIST)

Korea Electronics Technology Institute (KETI)

E-mail: Shin-Hyun Kim (kim.sh@kaist.ac.kr)

Contents

S1. Mechanochromic colloidal photonic crystals in literature

S2. Estimation of thickness of solvation layer

S3. Size distribution of silica particles

S4. Bragg diffraction in a swollen state

S5. Cross-section SEM images of the photonic films

S6. Uniformity of the swollen photonic film

S7. Optical response during swelling of the photonic composite in cubic shape

S8. Influence of silica particle size on the swelling ratio of photonic composite

S9. Multiple steps of swelling and polymerization

S10. Approximation of refractive-index contrast from lattice model

S11. Reversibility of mechanochromism

S12. Mechanical property of the swollen photonic film

S13. Description for Supporting Movies

S1. Mechanochromic colloidal photonic crystals in literature

Elastic colloidal photonic crystals exhibit shifts in diffraction wavelength under compression, driven by lattice deformation. Inelastic particles arranged in close-packed arrays have a limited ability to rearrange during macroscopic deformation due to interparticle interlocking. In contrast, inelastic particles in non-close-packed arrays can rearrange more freely while maintaining crystalline order. Table S1 summarizes the normalized peak wavelength shifts ($\Delta\lambda_{max}/\lambda_0$) and the fractional change in reflectivity at 10% strain ($\Delta R/R_0$) reported for mechanochromic colloidal photonic crystals in previous studies. Compared to these, our photonic films, with enhanced interparticle spacing, demonstrate superior performance in both $\Delta\lambda_{max}/\lambda_0$ and $\Delta R/R_0$.

Table S1. Comparison of the normalized peak wavelength shifts and the fractional change in reflectivity at 10% strain

No.	Type	λ_o (nm)	$\Delta\lambda_{max}$ (nm)	$\Delta\lambda_{max}/\lambda_o$	$\Delta R/R_0$	Reference
**1**	**Close-packed**	730	30	0.04	-	*Cell Rep. Phys. Sci.* **2023**, 4, 101490.
**2**		900	45	0.05	-	*J. Chem. Eng.* **2024**, 498, 155297.
**3**		680	80	0.11	-	*Nat. Mater.* **2006**, 5, 179.
**4**	**Non-close-pack**	525	65	0.12	0.24	*Adv. Funct. Mater.* **2014**, 24, 3197.
**5**		625	100	0.16	0.24	*Adv. Sci.* **2021**, 9, 2202897.
**6**		633	133	0.21	0.55	*Adv. Mater. Interfaces* **2022**, 9, 2200051.
**7**		658	140	0.22	-	*Appl. Nano Mater*. **2024**, 7, 11.
**8**		632	150	0.23	0.30	*Nat. Commun.* **2024**, 15, 1.
**9**		626	175	0.28	0.09	This work

S2. Estimation of thickness of solvation layer

When silica particles are dispersed in an acrylate resin, a solvation layer forms due to hydrogen bonding between the silanol groups on the particles surface and the acrylate groups in the resin and between the acrylate groups in the resins, making thick layer with multiple stacking of resin molecules, as shown in Figure S1a. This solvation layer consists of two distinct regions: a strongly bound inner layer and a loosely bound outer layers. The total thickness of the solvation layer (l_s) and the thickness of the immobile inner region (l_{in}) can be experimentally characterized from the crystallization threshold volume fraction (ϕ_{th}) and the transition volume fraction for the liquid-to-solid state (ϕ_{tr}), respectively. The volume fraction of spheres in a non-closed-packed face-centered cubic (fcc) lattice, denoted by $\phi(=\phi_{th})$, can be derived by analyzing a unit cell that contains 4 spheres:

$$\phi_{th} = \frac{4\times\frac{\pi}{6}D^3}{a^3} \ . \quad \text{(S1)}$$

Here, D represents the diameter of the spheres, and a is the lattice constant. From geometric considerations on the (111) plane, the center-to-center distance between two nearest neighbors, D_{cc}, is equal to $a/\sqrt{2}$. Therefore, the total solvation layer thickness (l_s) can be expressed:

$$l_s = \frac{1}{2}(D_{cc} - D) = \frac{1}{2}\left(\left(\frac{\pi}{3\sqrt{2}\phi_{th}}\right)^{\frac{1}{3}} - 1\right)D = \frac{1}{2}\left(\left(\frac{0.7404}{\phi_{th}}\right)^{\frac{1}{3}} - 1\right)D \ . \quad \text{(S2)}$$

In the silica-PEGPEA system, l_s is approximately 36.5 nm from D = 150 nm and ϕ_{th}= 0.225.[32] Based on this value, ϕ_{th} values are calculated as 0.248 for particles with a diameter of 166 nm, 0.276 for 185 nm, and 0.291 for 200 nm.

The liquid-to-solid transition occurs at volume at volume fractions below the maximum random close-packing limit of 0.64,[38] where particles adopt an amorphous arrangement rather than a crystalline structure. The thickness of the immobile inner region (l_{in}) can be estimated from the transition volume fraction (ϕ_{tr}):

$$l_s \sim \frac{1}{2}\left(\left(\frac{0.64}{\phi_{tr}}\right)^{\frac{1}{3}} - 1\right)D \ . \quad \text{(S3)}$$

For silica particles in a PEGPEA resin, l_s is approximately 3.8 nm.[38] With the thickness of the immobile region, ϕ_{tr} values are estimated as 0.552 for particles with a diameter of 150 nm, 0.567 for 185 nm, and 0.572 for 200 nm.

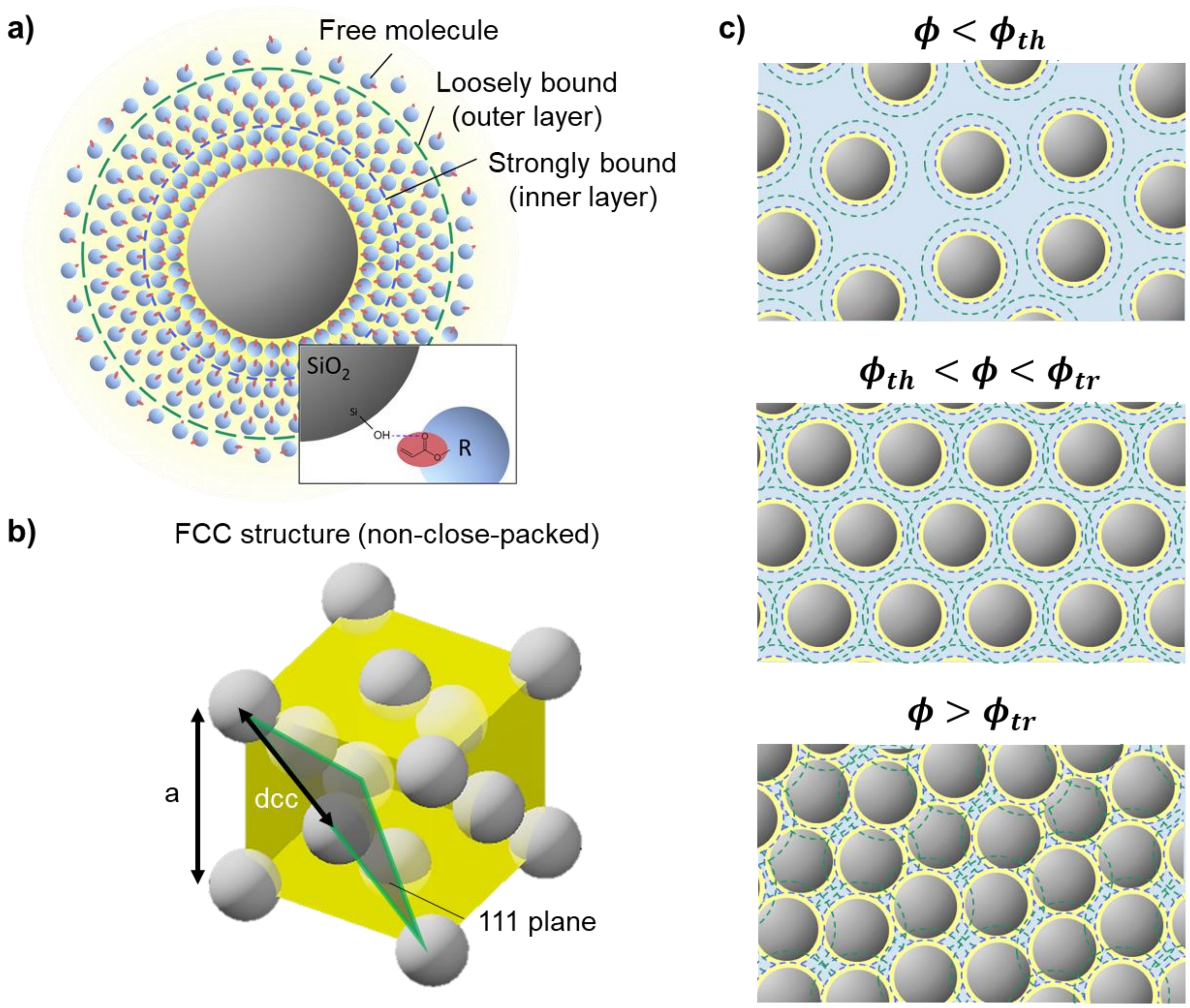

Figure S1. Solvation layer on the surface of the silica particle. a, b) Schematic illustration of the solvation layer, comprising a strongly bound inner layer and a loosely bound outer layer (a) and the unit cell of non-close-packed fcc lattice (b). (c) Particle arrangements depending the particle volume fractions: smaller than the threshold for crystallization (top), intermediate between the thresholds for crystallization and liquid-to-solid transition (middle), larger than the threshold for the transition (bottom).

S3. Size distribution of silica particles

The average diameter and coefficient of variation (CV) of silica particles were determined from the SEM image in Figure S2a. The particles exhibited an average diameter of 150 nm, with a CV of 4%, as shown in Figure S2b.

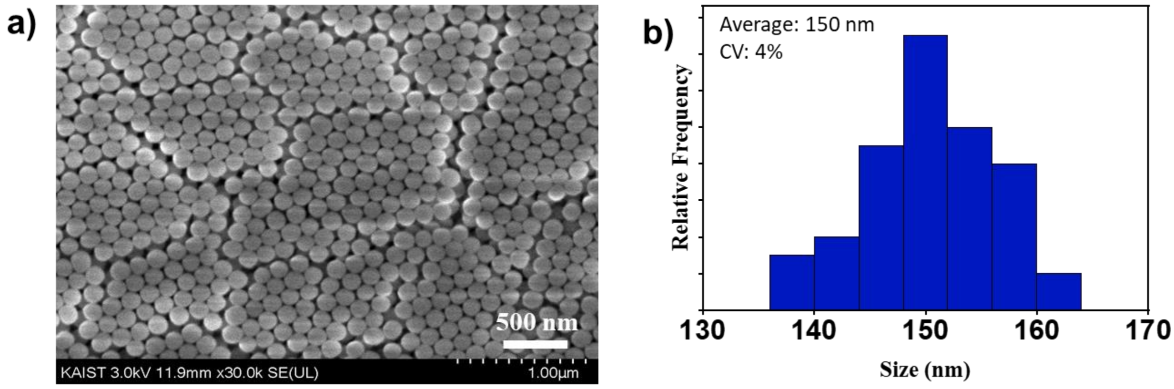

Figure S2. SEM image of silica particles and their corresponding particle size distribution.

S4. Bragg diffraction in a swollen state

As the photonic composite film swells, the lattice expands, leading to a red-shift in the stopband. The degree of expansion can be estimated by measuring the reflectance peak positions for pristine and swollen states. The initial diffraction wavelength of the photonic composite film can be described by Bragg's law:

$$\lambda_{111} = 2d_{111}n_{eff} = \left(\frac{\pi}{3\sqrt{2}\phi}\right)^{1/3}\left(\frac{8}{3}\right)^{1/2} Dn_{eff} \quad . \text{(S4)}$$

Upon swelling, the volume fraction of silica particles decreases to ϕ/α^3 and the lattice constant, d_{111} increases in proportion to the degree of lattice expansion, such that $d_{111} = \alpha d_{111,initial}$. Also, the effective refractive index of the swollen photonic composite is modified:

$$n_{eff} = \left(n_{silica}^2 \frac{\phi}{\alpha^3} + n_{mixture}^2\left(1 - \frac{\phi}{\alpha^3}\right)\right)^{1/2} . \quad \text{(S5)}$$

Consequently, the modified Bragg's law can be expressed as:

$$\lambda_{111} = 2d_{111}n_{eff} = \alpha\left(\frac{\pi}{3\sqrt{2}\phi}\right)^{1/3}\left(\frac{8}{3}\right)^{1/2} D\left(n_{silica}^2 \frac{\phi}{\alpha^3} + n_{mixture}^2\left(1 - \frac{\phi}{\alpha^3}\right)\right)^{1/2} . \quad \text{(S6)}$$

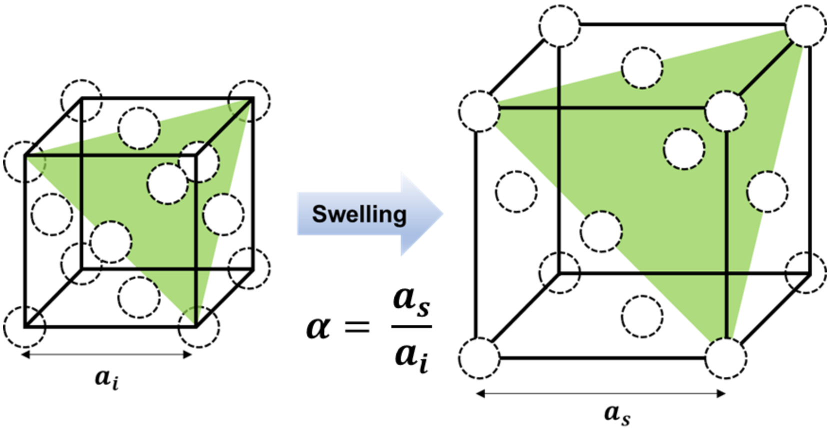

Figure S3. Schematic diagram of the fcc unit cell in the pristine (left panel) and the swollen states (right panel).

S5. Cross-section SEM images of the photonic films

To examine the increased distance between silica particles resulting from swelling, cross-sectional SEM analyses were performed on both unswollen and PEGPEA-swollen films as shown in Figure S4. The analysis revealed that silica particles were embedded within the pPEGPEA matrix, making it challenging to precisely measure the interparticle distance from the images. However, the hexagonal arrangement of the particles was preserved and the lattice expansion induced by swelling was distinctly observed, confirming the structural changes occurring during the swelling process.

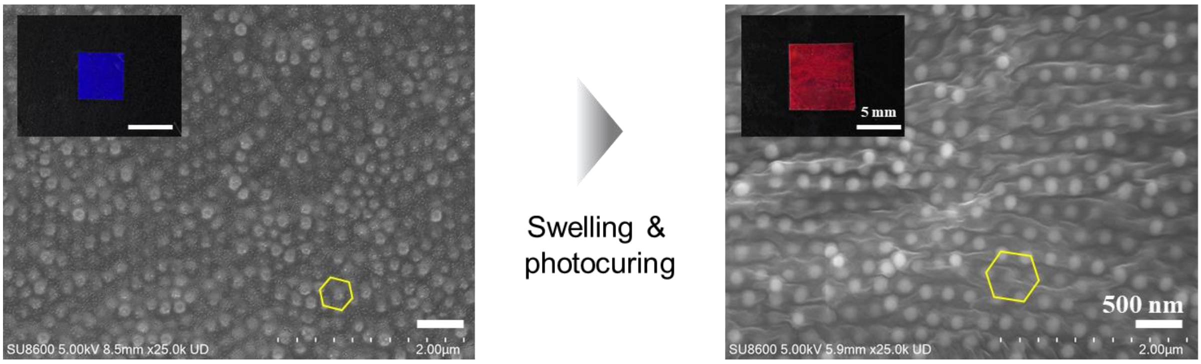

Figure S4. Cross section SEM images with photographs (inset) of unswollen (left) and swollen (right) photonic films

S6. Uniformity of the swollen photonic film

The film, with lateral dimensions of 5.4 cm × 3.7 cm, swelled uniformly, resulting in a consistently colored surface, as shown in Figure S5a. Optical microscopy images and reflectance spectra taken at 15 different locations across the film reveal consistent color, peak positions, and peak heights, as depicted in Figure S5b-d. This uniformity suggests that the swelling process maintains a homogeneous lattice structure, with no significant variations in optical properties.

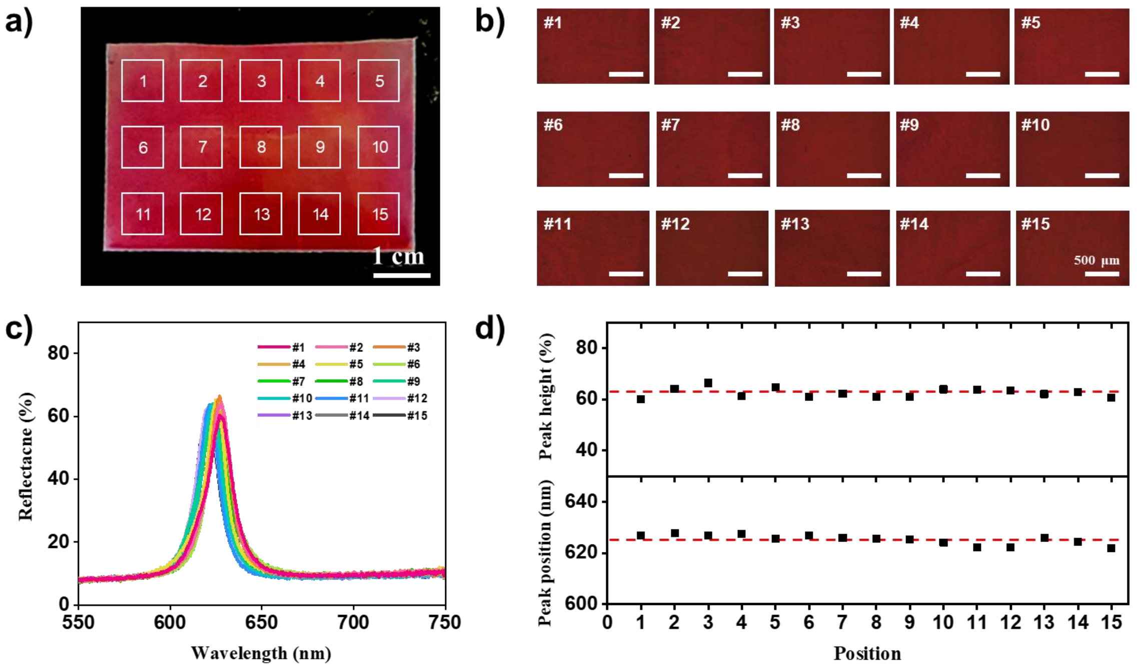

Figure S5. Uniform swelling of wide films. a) Photograph of the swollen photonic film after UV curing. b, c) Optical microscopy (OM) images and reflectance spectra of the film measured at 15 different positions. d) Peak height (top) and peak position (bottom) at the 15 different positions. Horizontal dotted lines represent the average values.

S7. Optical response during swelling of the photonic composite in cubic shape

To study the influence of geometry on swelling process, we prepared a cubic sample using silica particles with a diameter of D = 150 nm and the volume fraction at ϕ = 0.35. During the swelling process of the cubic-shaped photonic composite, the reflectance spectrum showed a gradual decrease in the intensity of the original peak at 465 nm, while a new peak emerged and redshifted to 710 nm, as illustrated in Figure S6. This behavior contrasted sharply with the film shown in Figure 2. Swelling initiates primarily at the surface and progresses slowly inward due to the limited diffusion rate of the solvent. Since the cubic structure has similar dimensions along all three axes, the expansion of the shell does not anisotropically compress the core. As a result, the original peak did not experience a blueshift. Additionally, the simultaneous redshift of the original peak and the blueshift of a new peak, observed in the film, did not occur in the cubic structure. The swelling ratio of the cubic sample, estimated from the original and redshifted peak positions, is 3.56, which is higher than that for the film, which is 3.06. The higher swelling ratio of the cubic sample is caused by higher volume fraction of the pPEGPEA matrix.

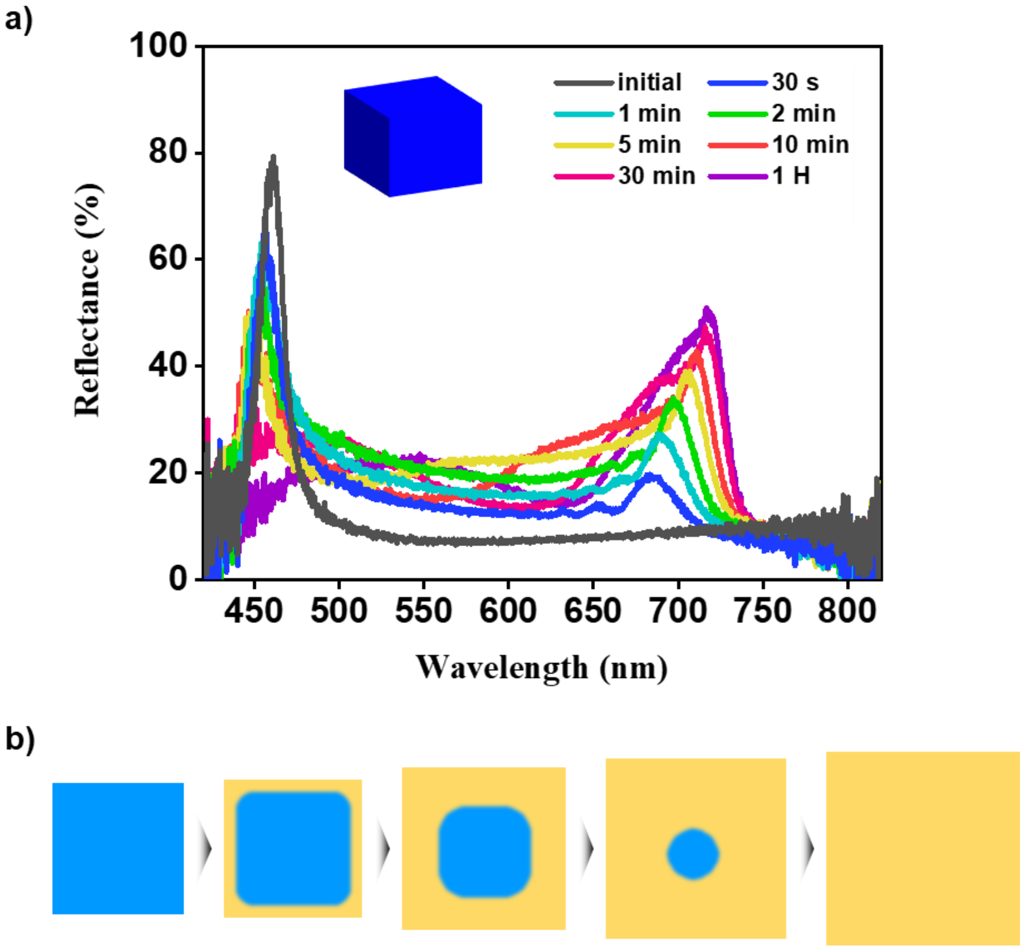

Figure S6. a) Reflectance spectra captured during swelling process for cubic-shape photonic composite with dimensions of 3 × 3 × 3 mm³. b) Schematic illustration depicting the swelling dynamics of the thin film.

S8. Influence of silica particle size on the swelling ratio of photonic composite

To investigate the effect of silica particle size on the degree of swelling, composite films were prepared using silica particles with diameters of 150, 185, and 200 nm, while maintaining a constant volume fraction of 0.40. The films underwent swelling and photopolymerization, and their reflectance spectra were measured, as illustrated in Figure S7b. From the original and redshifted peak positions, the swelling ratio and final volume fraction of silica particles were determined, as shown in Figure S7c. The results indicated that the swelling ratio and final volume fraction are consistent across all three particle sizes, with values of approximately 2.7 and 0.15, respectively. This suggests that the solvent selectively swells the polymer matrix without affecting the silica particles.

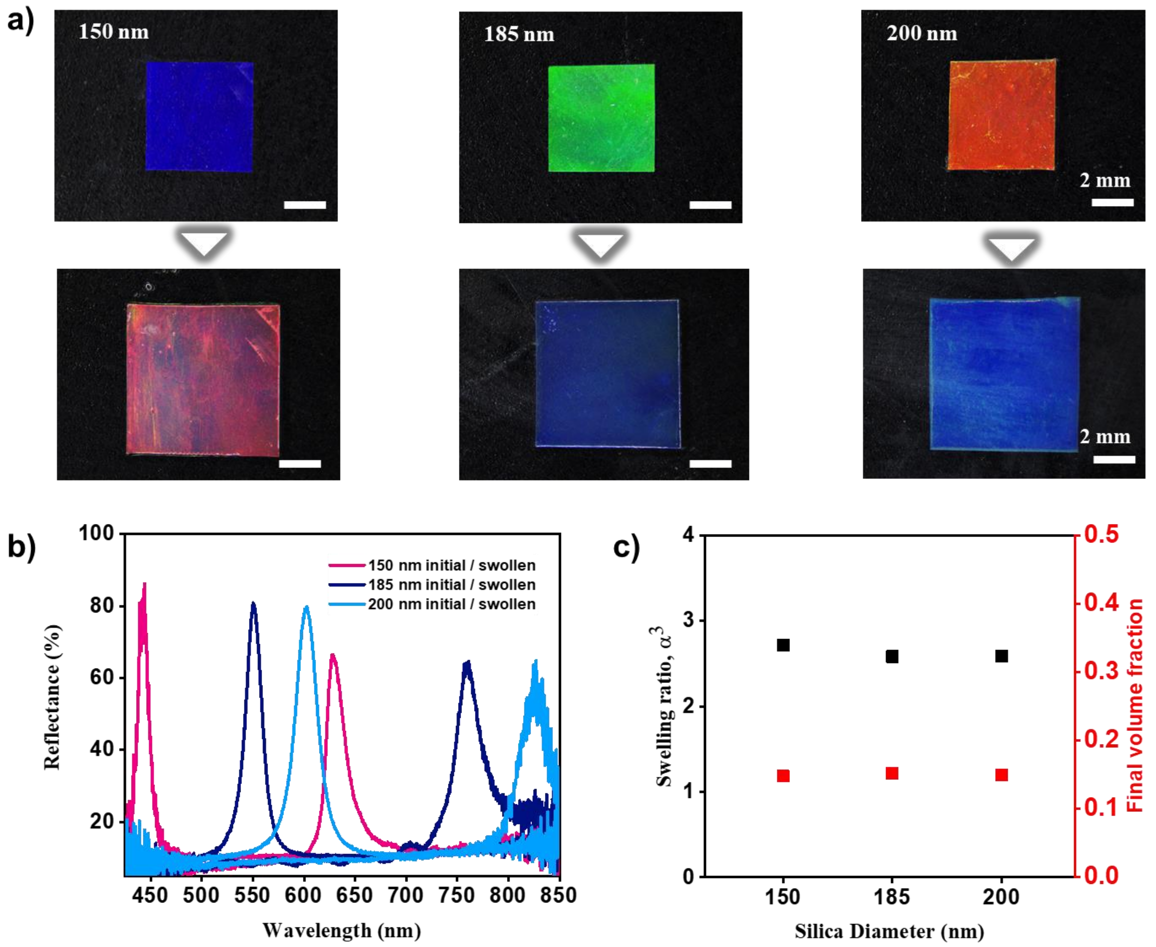

Figure S7. Negligible influence of particle size on degree of swelling. a, b) Sets of photographs (a) and reflectance spectra (b) of pristine and swollen films with varying silica diameters, as indicated. c) Swelling ratio and final volume fraction of silica particles in the swollen films for three different diameters of silica particles.

S9. Multiple steps of swelling and photopolymerization

The photonic composites can undergo multiple cycles of swelling and photopolymerization to further expand the lattice. To investigate this, we prepared four photonic composite films with varying crosslinking densities by adjusting the PEGDA concentrations to 0%, 5%, 10%, and 20%. The films were then immersed in PEGPEA for swelling and subsequently photopolymerized. This process was repeated for a second and third swelling cycle. A more pronounced redshift was observed in films with lower PEGDA concentrations as shown in Figure S8. With each successive swelling, the peak continued to redshift, as demonstrated.

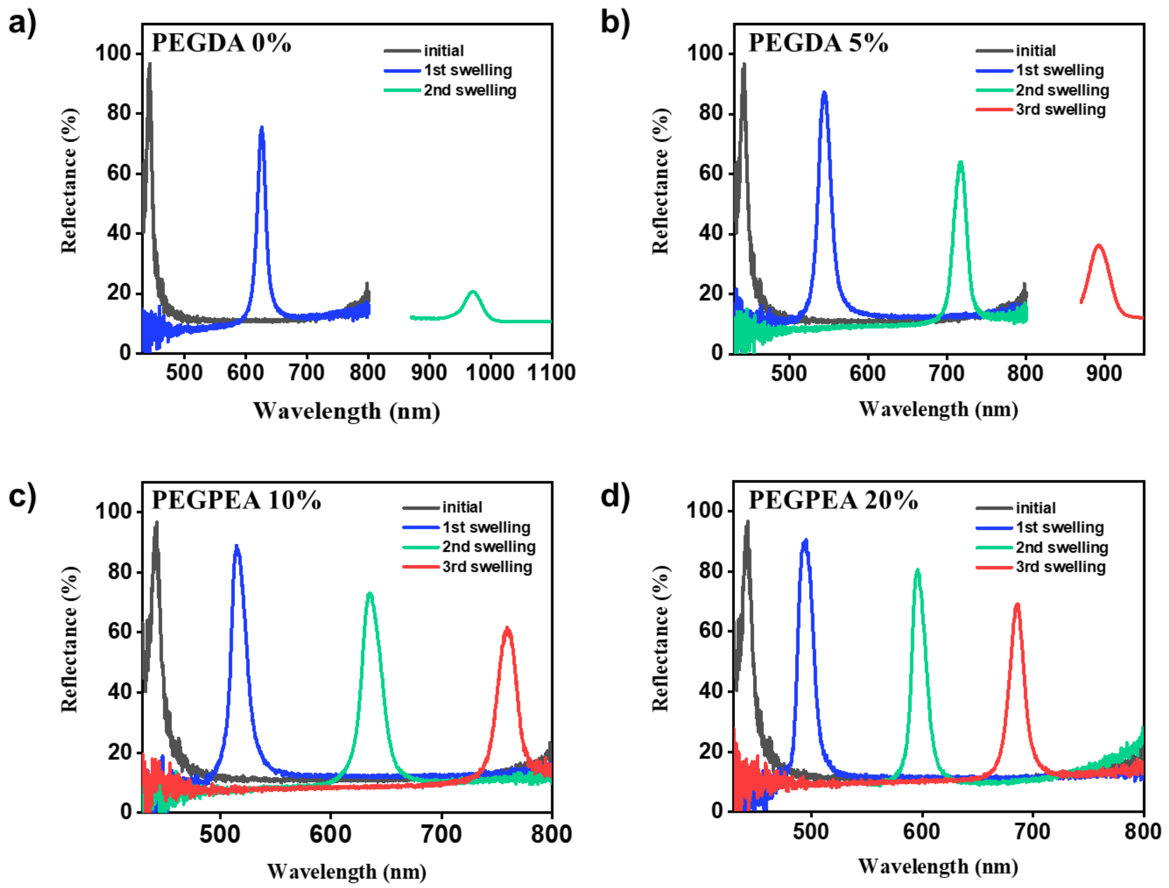

Figure S8. The reflectance spectra for multiple steps of swelling and photopolymerization of photonic composites with four different PEGDA concentration: (a) 0%, (b) 5%, (c) 10%, and (d) 20%.

S10. Approximation of refractive-index contrast from lattice model

During the swelling process, the distance between particles increases while the crystalline array remains intact. We model the lattice expansion, as shown in Figure S9a. The reflectivity depends on the refractive index contrast between two repeating layers: particle-rich and particle-depleted regions. The effective refractive indices of these two layers, which have the same thickness, are calculated using the Maxwell-Garnett approximation based on the model structure. The refractive index contrast decreases as the final volume fraction of silica particles reduces along with the degree of swelling, as shown in Figure S9b.

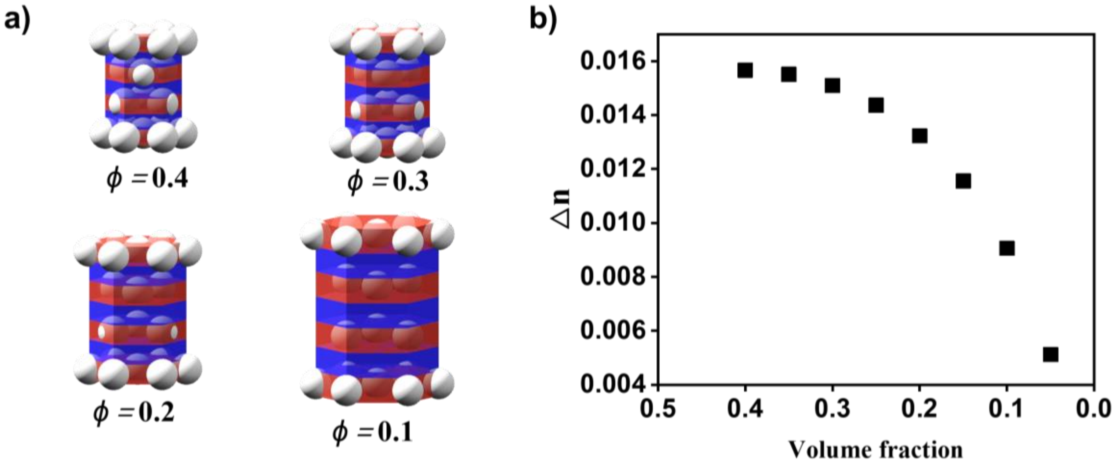

Figure S9. a) Lattice models illustrating structural expansion to have various final volume fractions of silica particles, as indicated. b) Refractive index contrast between particle-rich and particle-poor layers as a function of the final volume fraction of silica particles.

S11. Reversibility of mechanochromism

To evaluate the reversibility of the color changes in swollen photonic films, cycling tests were performed as shown in Figure 10. Throughout 20 cycles of compression and relaxation, ranging from 0% to 20% strain, no significant changes in reflectance and λ_{max} were observed. Because the colloidal particles remain in their original arrangement during deformation, no residual stress occurs once the strain is released, enabling the film to fully recover its reflectance and λ_{max}. Furthermore, the color change and recovery occurred instantaneously, with no detectable delay.

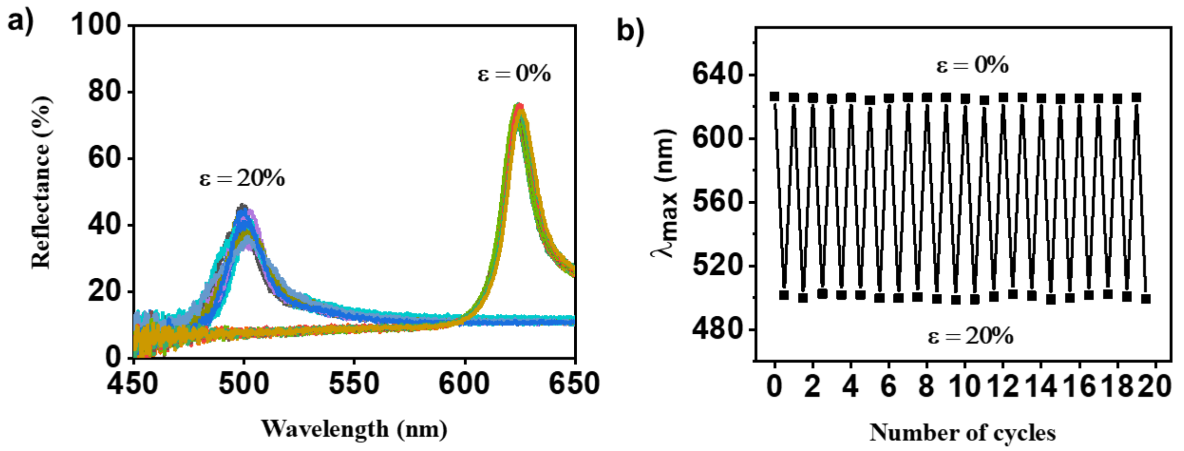

Figure S10. Reversibility of the spectrum shift under compression and relaxation cycles. a, b) The reflectance spectra and the change of λ_{max} of swollen photonic film during 20 cycles of compression to $\varepsilon = 20\%$ and relaxation to $\varepsilon = 0\%$.

S12. Mechanical property of the swollen photonic film

To characterize the mechanical properties of the photonic film before and after swelling, compression tests were performed. The modulus of the swollen film was comparable to that of the unswollen one for compressive strains smaller than 0.1 but became larger at compressive strains exceeding 0.1 even though the volume fraction of silica particles decreased from 0.40 to 0.14 due to swelling with PEGPEA, as shown in Figure S11. Contrary to the initial expectation that the modulus would decrease due to the reduced particle volume fraction, the increased modulus after swelling is likely attributed to the partial stretching of the physically entangled pPEGPEA chains during the swelling process, which enhances stiffness of the film.

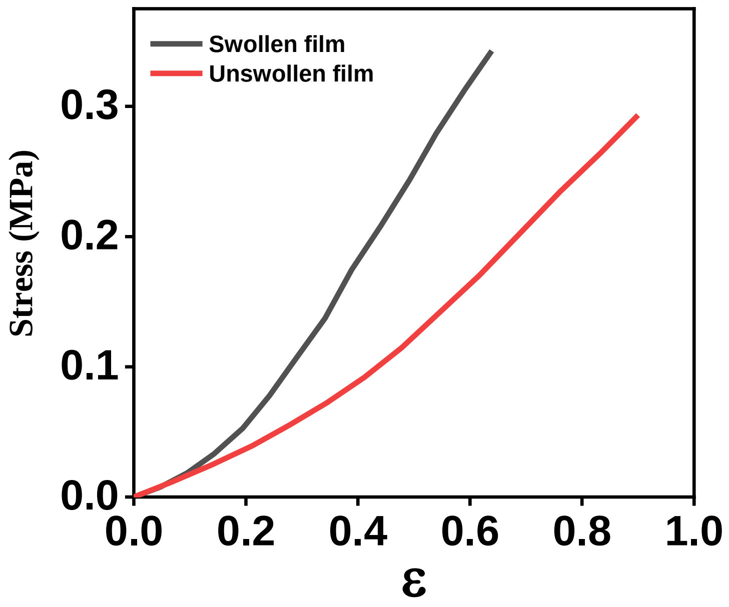

Figure S11. Strain-stress curves of unswollen and swollen photonic films during compression.

S13. Description for Supporting Movies

- **Movie S1:** Swelling of a blue film in PEGPEA observed under a stereomicroscope. The video is accelerated 100-fold in the first segment (corresponding to 5 minutes in real time), 500-fold in the second segment (corresponding to 115 minutes in real time), and 1000-fold in the third segment (corresponding to 240 minutes in real time). A ring pattern emerges during the swelling process, which is a visual artifact caused by minor distortions in the film.
- **Movie S2**: Reversible color change of swollen photonic film during compression and relaxation.
- **Movie S3**: Development of ‘PHOTONIC CRYSTAL’ color pattern by compressing photonic film with a corresponding stamp.
